# Pathway Bridge Based Multiobjective Optimization Approach for Lurking Pathway Prediction

**DOI:** 10.1155/2014/351095

**Published:** 2014-04-16

**Authors:** Rengjing Zhang, Chen Zhao, Zixiang Xiong, Xiaobo Zhou

**Affiliations:** ^1^Electrical and Computer Engineering Department, Texas A&M University, College Station, TX 77840, USA; ^2^Radiology Comprehensive Cancer Center Cancer Biology, Wake Forest University, Winston-Salem, NC 27103, USA

## Abstract

Ovarian carcinoma immunoreactive antigen-like protein 2 (OCIAD2) is a protein with unknown function. Frequently methylated or downregulated, OCIAD2 has been observed in kinds of tumors, and TGF**β** signaling has been proved to induce the expression of OCIAD2. However, current pathway analysis tools do not cover the genes without reported interactions like OCIAD2 and also miss some significant genes with relatively lower expression. To investigate potential biological milieu of OCIAD2, especially in cancer microenvironment, a nova approach pbMOO was created to find the potential pathways from TGF**β** to OCIAD2 by searching on the pathway bridge, which consisted of cancer enriched looping patterns from the complicated entire protein interactions network. The pbMOO approach was further applied to study the modulator of ligand TGF**β**1, receptor TGF**β**R1, intermediate transfer proteins, transcription factor, and signature OCIAD2. Verified by literature and public database, the pathway TGF**β**1- TGF**β**R1- SMAD2/3- SMAD4/AR-OCIAD2 was detected, which concealed the androgen receptor (AR) which was the possible transcription factor of OCIAD2 in TGF**β** signal, and it well explained the mechanism of TGF**β** induced OCIAD2 expression in cancer microenvironment, therefore providing an important clue for the future functional analysis of OCIAD2 in tumor pathogenesis.

## 1. Background

Tumor microenvironment has been largely studied as a dynamic system to define the behaviors of cancer. This system is orchestrated by cytokines, growth factors, inflammatory cells, cancer cells, stroma, as well as the extracellular matrix [1]. Tumor-associated fibroblasts (TAFs) are major elements of tumor stroma and have been shown to play an important role in tumor growth and progression. Epithelial-to-mesenchymal transition (EMT) is a major source of TAFs. In tissue fibrosis, it is well-established that epithelial cells contribute to the accumulation of fibroblasts by undergoing EMT in response to stimuli from the microenvironment [2]. TGF*β* remains among the key factors responsible for the recruitment of Tumor Associated Fibroblasts (TAFs) and induction of EMT. TAFs, meanwhile, strongly contribute to the production and activation of TGF*β* in the activated stroma and thereby generate the autocrine feed-forward loop that is characteristic for persisting fibroblasts activities [3]. However, the exact regulation between TGF*β* signals and TAFs in tumor microenvironment is yet to be completely understood.

OCIAD2 was originally immunoscreened from ascites of a patient with ovarian cancer and was found to be an immunoreactive antigen [4]. However, the function of OCIAD2 protein, involved pathways, and molecular mechanisms has never been reported. Based on our preliminary data analysis, we hypothesized that human OCIAD2 represents a potential tumor suppressor gene in some tumor types and its dysregulation is involved in TGF*β* regulated signaling in tumor microenvironment for the following reasons: (1) high-throughput profiling data and public database analyses showed that OCIAD2 is frequently methylated and/or downregulated in some kinds of cancers [5–7]; (2) GEO database revealed that the expressions of OCIAD2 are induced by TGF*β* signal in pancreatic (GSE23952), lung adenocarcinoma (GSE17708), and ovarian cancer cells (GSE6653); (3) moreover, a computational analysis with TCGA database revealed that methylation site of OCIAD2 is top-ranked signature in ovarian Metastasis-Associated Fibroblasts (MAFs) [8]. This evidence indicated a potential biological milieu of OCIAD2. We hereby speculate that downregulated OCIAD2 expression in tumor microenvironment facilitates deregulated TGF*β* signaling. As a consequence of these changes, tumor cells escape immunosurveillance and exaggerate tumor progression and metastatic spread.

To predict molecular network of OCIAD2 in TGF*β* regulated tumor microenvironment, a nova pathway analysis approach with bioinformatics methods has been developed. Current signal analysis methods typically have three steps: build literature based preliminary signaling pathways model; generate gene expression experimental data; detect the shortest path as the specific signal and verify biological meaning. Pathways consisting of highest differentially expressed genes and reported interactions would be shown as the results in this kind of pathway study methods. However, not all the targets or receptors of ligands are with top expression changes; that is, TGF*β* regulates numerous other growth factors positively and negatively, some of which are not the most obviously changed ones but still give response to the stimulation of TGF*β*. Moreover, new genes with seldom previous reports, such as OCIAD2, cannot be included in any pathways because of the lack of known interactions with other proteins. To study the mechanism of OCIAD2 changes induced by TGF*β* stimulation in cancer cell lines, a new approach to inferring the signaling paths based on the pathway bridges between the stimulant TGF*β* and its target gene OCIAD2 using the multiobjective optimization approach, named pbMOO, was developed. Pathway Bridge was defined as a subset of protein interactions network that consisted of clustering loop motifs with extremely high frequency occurring in cancer related processes than by chance. All four-vertex motifs, among which the triangle and rectangle were shown with significantly higher occurrences than randomized ones, were detected from a network generated from HPRD database with 12794 proteins and 39031 interactions. Rather than traversing the entire protein interaction network with enormous nodes and edges, all the loop motifs were clustered as a “Pathway Bridge” between TGF*β* signaling pathway and cancer signaling pathway. Relatively, the time saving approach returned to highly reliable protein paths only by searching connecting nodes on the bridge. Moreover, motifs on the bridge were concentrated on cancer related processes, which guaranteed that the nodes chosen for the path are specified for cancer microenvironment. Then, the cost of a protein path was defined by gathering up the cost of each edge, which is the* P* value sum of two interacted protein nodes. According to the property of *P* value, the path cost is the probability of obtaining a path whose cost is no more than the one that was actually observed. Hence, the reliability of a predicted pathway can be represented by its cost.

177 transcription factors of Homo sapiens were analyzed from Transcriptional Regulatory Element Database [9], and androgen receptor (AR) was discovered as the most credible transcription factor of OCIAD2. Applied the approach on GSE42357 and GDS3634 expression data from NCBI, and the paths with the lowest cost were picked out as the responsible possible molecular mechanisms between TGF*β* and OCIAD2 in hepatocellular carcinoma (HCC) samples and prostate cancer cell lines. After verifying the biological meaning of the low-cost paths, the signal TGF*β*1- TGF*β*R1- SMAD2/3- SMAD4- AR-OCIAD2 was discovered and explained TGF*β*'s stimulation on OCIAD2 expression in cancer.

## 2. Methods

**Figure 1:**
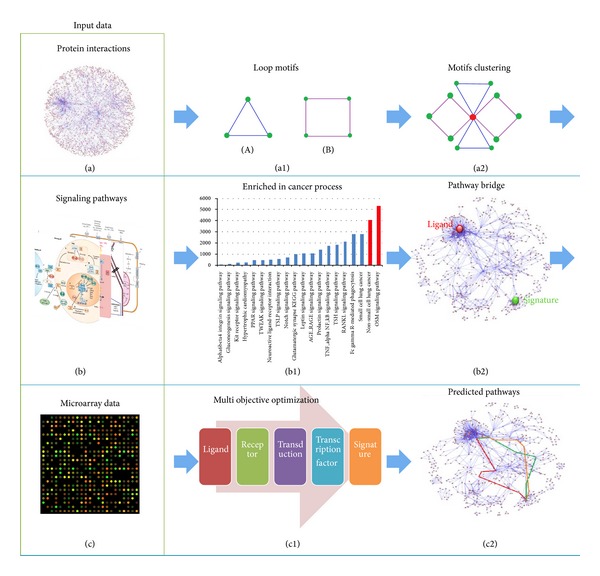


In order to see how the signature gene is enrolled in the ligand stimulation signal, a pathway bridge based multiobjective optimization approach (pbMOO) was designed and summarized, as shown in Figure 1. Three kinds of data were chosen as the initial input data: protein interactions (Figure 1(a)) from HPRD [10] were applied for building the entire protein-protein interactions (PPI) network; signaling pathways (Figure 1(b)) from both KEGG [11] and IPA [12] were selected as the background pathway library based on which the pathway bridges were constructed; groups of microarray data (Figure 1(c)) were used for assigning the path cost in the optimization problem and presenting the correlation between genes. Calculated by FANMOD [13], loop motifs (Figure 1(a1)), which were the higher frequency occurring subnetworks out of the entire PPI network, were shown to be enriched in cancer and related pathways. Searching on the pathway bridge (Figure 1(b2)), which was defined as a set of loop motif clusters (Figure 1(a2)) connecting ligand and signature genes, a multiobjective optimization problem was solved by finding the pathways with the lowest path cost that was assigned by gene expression *P* value. When multiple experimental gene expression data were used, the cost of each path was then defined as the summation of average *P* value of connected genes in the optimization problem. Then the modular study (Figure 1(c1)) was applied on the calculated results of the optimization problem. Finally, the integrated signals, which began with ligand and its receptor, passing through transduction proteins and targeting transcription factor and finally the signature, were output as the most reliable predicted pathways (Figure 1(c2)) explaining how the ligand changes affected the signature.

### 2.1. Cell Lines and Drug Treatment

Hep-3B and Du-145 were obtained from American Type Culture Collection. All cell lines were cultured in DMEM with 10% fetal bovine serum (FBS) and antibiotics. TGF*β*1 (R&D Systems, Minneapolis, MN) were applied at concentrations of 5 ng/mL. TGF*β*R inhibitor LY2109761 were purchased from Selleck Chemicals LLC (Houston, TX), using 2 *μ*M. For the drug treatment, human liver and prostate cancer cell lines, Hep-3B and Du-145 were treated with 5 ng/mL TGF*β*1, 2 *μ*M LY2109761, and combination for 24 hours in serum free media, and OCIAD2 mRNA levels were determined by quantitative real-time RT-PCR analysis.

### 2.2. RNA Extraction and Quantitative Real-Time PCR

Total RNAs were isolated from tumor cells using TRIZOL reagent (Life Technologies, USA) following the manufacturer's recommendations. RNA concentration and purity were determined by measuring absorbance at 260 and 280 nm with a NanoDropTM 1000 Spectrophotometer (Thermo Scientific, USA). cDNA synthesis was performed with Superscript III reverse transcriptase kit (Life Technologies, USA). Quantitative real-time reverse-transcription polymerase chain reaction (RT-PCR) was performed using an Applied Biosystems 7300 Sequence detection system (Applied Biosystems, Life Technologies, USA). The primer sets of OCIAD2 are described below: 5′-TGCGAGAATGTCAGGAAGAA-3′ and 5′-AAATCCCAAGAGACCAGCAA-3′.

### 2.3. Motif Detection of Protein-Protein Interaction Network

“Network Motifs” [14] are interconnected patterns (subgraphs) with significantly higher occurring in complicated networks than in randomized ones. In biological networks, almost all of the four vertex motifs were combinations of smaller motifs [15], and loop-structural motifs have been proved to be enriched in a protein-protein interaction (PPI) network generated from PPI database, that is, HPRD. As a literature-collected public database, HPRD has 12794 proteins and 39031 pairs of interactions for 9605 of them. The sufficient data capacity helps a lot on unclear reciprocities prediction.

In this paper, the outstanding tool Fast Network Motif Detection (FANMOD) [13] was applied to census four-vertex subgraphs in undirected PPI network by using the Randomized Enumeration (RAND-ESU) algorithm.

**Figure 2:**
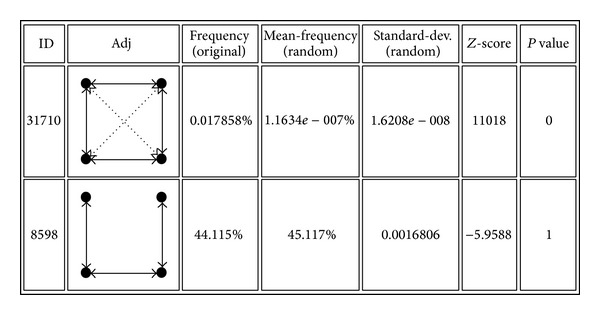


Motifs detection results from PPI network were shown as an example in Figure 2 and detailed in Supplementary 1.1 in Supplementary Material available online at http://dx.doi.org/10.1155/2014/351095. Motifs ID lied in column one and adjacency matrix was presented in the second column; Frequency was the probability of each motif in original PPI network, and Mean-Frequency was of the motif that occurred in random networks; the standard deviation from the mean frequency was listed in the fifth column;* Z*-score meant the value of the difference of two frequencies divided by the standard deviation; and* P* value was the difference of motif numbers between random networks and original one then divided by the total number of random networks. ID 8598 in Figure 2 had relatively higher occurring frequency in random networks than the original one, and thus its *Z*-Score was negative and* P* value was large, which indicated that the chain-looked structure was really normal among the entire PPI network; on the contrary, ID 31710 was more special in the original network structure than randomly showing that the combination of triangle and square subnetwork was enriched among PPI network. Similarly, motifs ID 4382, recurring much more often than random chosen subnetwork, were with negative *Z*-Score and large* P* value; ID 13278, ID 4958, and ID 27030 motif structures were also enriched from randomized network and obtain positive *Z*-Score and relatively smaller* P* value. The whole results were listed in the supplementary. The outcome suggests loop-structural motifs, that is, shapes like triangle, spoon, and square, which are special patterns with high occurrences in protein interactions network.

**Figure 3:**
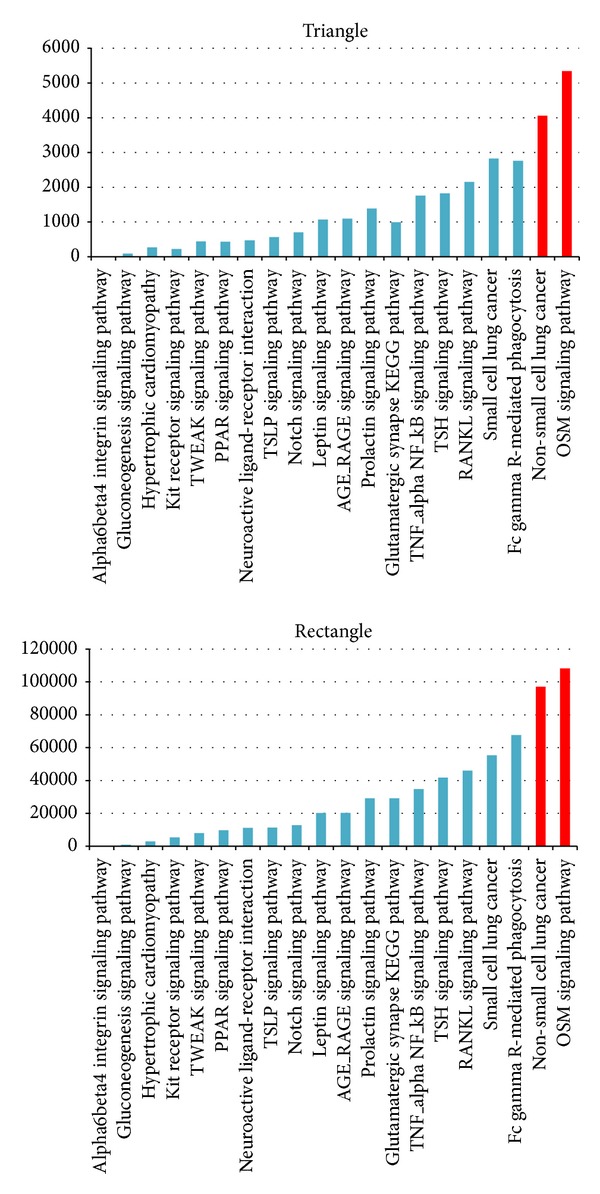


20 signaling pathways derived from KEGG [11] were analyzed for motifs distribution. Results in Figure 3 showed that proteins on looping motifs are mainly from cancer and correlated signal pathways, in other words, motifs with loop structure are enriched in 14 types of carcinomatosis and related signaling pathways, such as cell cycle signaling pathway and immune system signaling pathways.

### 2.4. Motifs Clustering and Enrichment in Cancer Related Signaling Pathways

**Figure 4:**
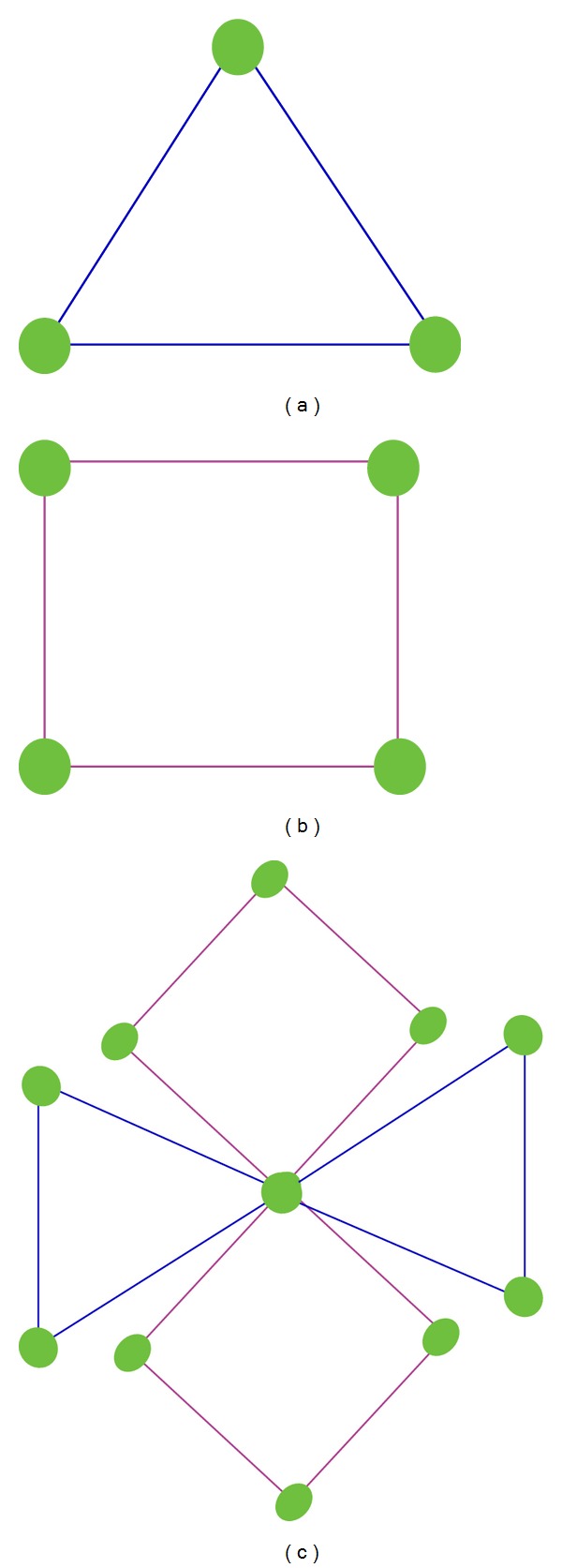


Loop-shaped motifs with no more than four vertices have the only two specific possibilities—triangle and square. Motifs Cluster (MC) is defined as converged cyclic motifs that share at least one protein. The common protein is called Center Point (CP), which is the identifier for distinguishing different motifs clusters. The toy model was showed in Figure 4.

Since looping motifs were proved to be occurring much more often in cancer and related signaling pathways, they can be treated as a bridge to link up cancer and its kinship pathways, which would provide a characteristic group of candidate protein interactions for future unclear links forecast.

Let *P*_1_ be a chosen cancer signaling pathway, and let *P*_2_ be a cancer-related signaling pathway. {MC_1_^*P*_1_*P*_2_^, MC_2_^*P*_1_*P*_2_^,…, MC_*n*_^*P*_1_*P*_2_^} are the total *n* motif clusters between *P*_1_ and *P*_2_, and thus |MC^*P*_1_*P*_2_^ | = *n* by virtue of the number of identifiers |CP^*P*_1_*P*_2_^ | = *n*, where |·| denotes the number of elements in a set. In order to evaluate the enrichment of MCs lying between *P*_1_ and *P*_2_, *P* value was introduced as the probability of obtaining a larger number of MCs for a pair of randomly chosen protein sets, keeping the same sizes with *P*_1_ and *P*_2_ and the capacity of intersection, than for *P*_1_ and *P*_2_. Consider the following:

(1) $$\begin{array}{c} p=\text{prob}\left\{n^{\prime }>n\;|\;n^{\prime }=\left|\text{M}{\text{C}}^{S_{1}S_{2}}\right|,n=\left|\text{M}{\text{C}}^{P_{1}P_{2}}\right|\right\}, \end{array}$$

where *S*_1_ and *S*_2_ are random protein sets picked out from entire proteins of HPRD database with the same size as *P*_1_ and *P*_2_; that is, |*S*_1_ | = |*P*_1_| and |*S*_2_ | = |*P*_2_|, and satisfy |*S*_1_ ⋂ *S*_2_ | = |*P*_1_ ⋂ *P*_2_|. Repeating the sampling for 1000 times, a random distribution *f* for the 1000 numbers of MCs can be generated. The complementary set of cumulative probability density function *F*(|MC^*P*_1_*P*_2_^|) interprets the chances that a stochastic pair of protein sets has quantity of MCs which are being the bridges between them rather than the two chosen pathways, which is indeed motif clusters' *P* value. Consider the following:

(2) $$\begin{array}{c} p=F^{\prime }\left(\left|\text{M}{\text{C}}^{P_{1}P_{2}}\right|\right)=1-F\left(\left|\text{M}{\text{C}}^{P_{1}P_{2}}\right|\right). \end{array}$$

MCs connecting *P*_1_ and *P*_2_ are enriched if the *P* value is tiny, indicating that the bridging MCs linking up two protein sets are the main substructure of cancer signaling path *P*_1_ and cancer enrolled signaling path *P*_2_. Comparing with searching the enormous and complex integrated PPI network, the enriched MCs bridge efficiently limits and specializes the traversing range for forecasting uncertain protein paths, which increases the calculation speed thoroughly.

**Figure 5:**
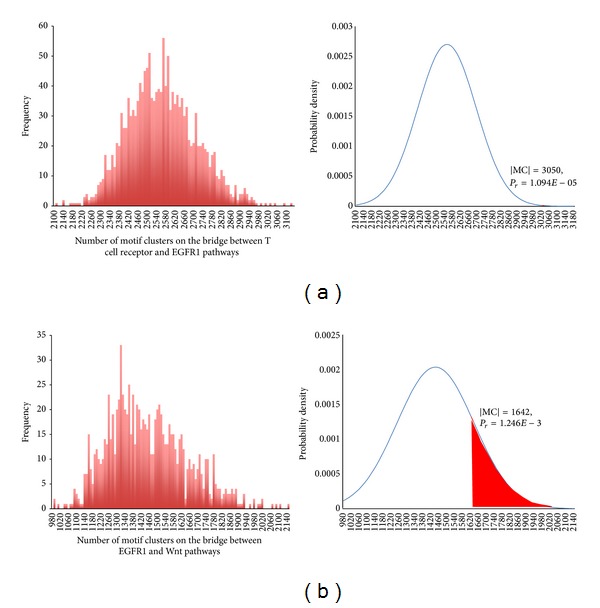
(a) Enriched Motif Clusters between EGFR1 and T cell receptor pathways. (b) Less Enriched Motif Clusters between EGFR1 and Wnt pathways.

MCs' *P* value for combinations of different types and subtypes of carcinomatosis and involved signaling pathways were calculated and exampled in Figure 5. MCs bridges that have *P* value less than 0.01 were chosen to be the candidate subnetwork, from which ill-defined protein pathways would be selected.

### 2.5. Ill-Defined Protein Pathways Prediction

If changing condition of a protein A results in the upregulated or downregulated protein B, while A and B have neither direct interaction with each other nor indirect upstream and downstream relationship on any authentic signaling pathway, the underlying protein pathways for them could be detected on those MCs enriched pathway bridges. An optimization model, which is described as follows, was employed to acquire high-confidence potential protein pathways:

(3) $$\begin{array}{c} f\left(x\right)=\underset{\vec{x}\in \{\text{M}{\text{C}}_{1}^{P_{1}P_{2}},\text{M}{\text{C}}_{2}^{P_{1}P_{2}},\ldots ,\text{M}{\text{C}}_{n}^{P_{1}P_{2}}\}}{\text{argmin}}\overset{N}{\underset{i=1}{\sum }}x_{i}\cdot \text{DE}{\text{S}}_{i}+\lambda \overset{N}{\underset{i=1}{\sum }}x_{i},\\ \text{s}.\text{t}.\;\{\begin{array}{cc} 2\leq \overset{N}{\underset{i=1}{\sum }}x_{i}\leq 7, & \\ i=1,2,\ldots ,N, & \\ \lambda \in \left|R\right|, & \end{array} \end{array}$$

where *N* is the total number of proteins pertaining to MCs bridge for the selected pair *P*_1_ and *P*_2_. $\vec{x}=\{x_{1},x_{2},\ldots ,x_{N}\}$ is protein path vector implying which element was contributed to the path—if protein *i* was taken count into the lurking protein path, then *x*_*i*_ = 1; otherwise, *x*_*i*_ = 0. Differential expression score (DES) was defined by each gene's *P* value from Student's *t*-test of gene expression experiment data in two conditions. The larger the *P* value, the larger the DES, and the less reliable the data. Thus, minimizing the first part of the objective function ∑_*i*=1_^*N*^ *x*_*i*_ · DES_*i*_ could ensure the maximization of the reliability of the predicted protein paths. The length of protein pathway; that is, ∑_*i*=1_^*N*^ *x*_*i*_, is an integer in the range of [2,7], which was decided by the fact that MCs were composed of looping structures up to 4 vertices. At this point, the latter part of the objective function took the responsibility of controlling the length of analyzed protein pathway with the aid of distinct settings of nonnegative parameter *λ*. |*R*| is the absolute value of real number. Large *λ* was made for limited proteins and short connections, and optimization result was free to rope in proteins when *λ* = 0.

The optimization function was solved by the shortest path package in R, where Dijkstra's algorithm was employed and the length controlling parameter *λ* was set as zero to gain all the possible predictions. The solution of the optimization function was the optimal protein path vector $\vec{x}$ of *N* elements *x*_*i*_, representing protein *i* was in the lowest-cost path or not.

### 2.6. Interacted Pairs Inference for Protein without PPI

For those proteins that have no canonical protein interaction supported, gene expression data was conduced to providing indistinct mutual effects and pointing out candidate proteins with which the separated proteins were closely bound up by the correlation between the pairs of genes.

### 2.7. Multiple Microarray Data Based Differential Expression Score

As a matter of fact, the* P* value of experimental gene expression data may vary a lot by different experiment designs and operators, and a good inferred protein path is the one which gets rid of the destabilizing factors. Thus, multiple microarray data sets were employed here for error deduction.

## 3. Results and Discussion

### 3.1. Dysregulation of OCIAD2 in Different Cancers and Its Induction by TGF*β* in HCC and PC Cells

**Figure 6:**
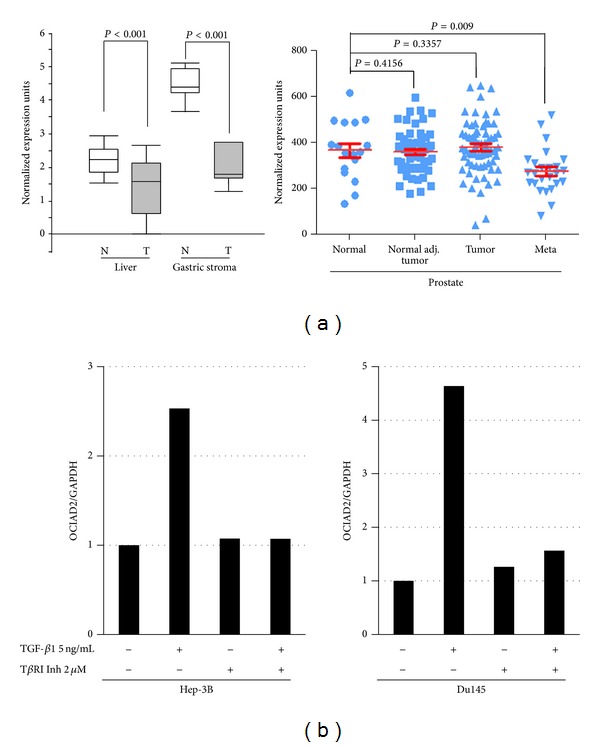
Expression of OCIAD2 and its induction by TGF-*β*.

To determine the OCIAD2 expression, different available microarray studies were analyzed by the Oncomine database and GEO gene microarray data analysis tools. A significant downregulation of OCIAD2 mRNA expression was found in liver cancer and gastric stroma carcinoma tissues (*P* < 0.001 in both cases) (Figure 6; Left) based on Oncomine database analysis. The result indicated that OCIAD2 expression is in metastatic prostate tissues, but not in primary tumor tissues, which is clearly lower than normal prostate gland (*P* = 0.009) (Figure 6; Right; GSE6919). Frequently downregulated OCIAD2 expressions are also observed in CLL and malignant pleural mesothelioma [5, 6]. In glioblastoma, OCIAD2 expression is being silenced via DNA methylation mechanism [7]. With the suggestion of the fact that OCIAD2 was substantially unregulated in TGF*β*1 treated pancreatic (GDS4106), lung (GSE17708), and ovarian (GSE6653) cancer cells, we have tested the possibility of OCIAD2 expression inducted by TGF*β* in HCC and PC cells. Human HCC and PC cell lines, Hep-3B and Du-145, were treated with 5 ng/mL TGF*β*1, 2 *μ*M LY2109761, and combination for 24 h in serum free media, and OCIAD2 mRNA levels were determined by quantitative real-time RT-PCR analysis. OCIAD2 mRNA has increased 2.5- and 4.6-fold in Hep-3B and Du-145 cells by TGF*β*1 treatment, respectively. This induction was totally suppressed by TGF*β*1 receptor inhibitor LY2109761 (Figure 6(b)).

### 3.2. Potential Protein Pathway Prediction

Remarkable gene array profiles from GEO database indicated the expression of OCIAD2 in several kinds of cancer; that is, GDS3634 showed that OCIAD2 was obviously unregulated in prostate cancer cell line transfected with 20 nM miRNA Presursor Molecules miR-205. MiR-205 is selectively downregulated in metastatic breast and prostate cancer and suppresses metastatic spread of a human breast cancer xenograft in nude mice. In addition to its function in the regulation of EMT, the loss of miR-205 in prostate cancer also reduced some tumor suppressor genes' expression.

Mesenchymal stem cells (MSC), like other bone marrow-resident cells, have the capacity to differentiate into fibroblast-like cells that have been variably referred to as myofibroblasts, tumor-associated fibroblasts (TAF), fibrocytes, or pericytes within the tumor microenvironment [16]. Therefore, pbMOO approach was applied on both prostate cancer cell line GDS3634 and liver cancer associated mesenchymal stem cells GSE42357 gene expression data to study the possible molecular path involved in OCIAD2 by TGF*β* stimulation.

#### 3.2.1. Pathways in Prostate Cancer

**Figure 7:**
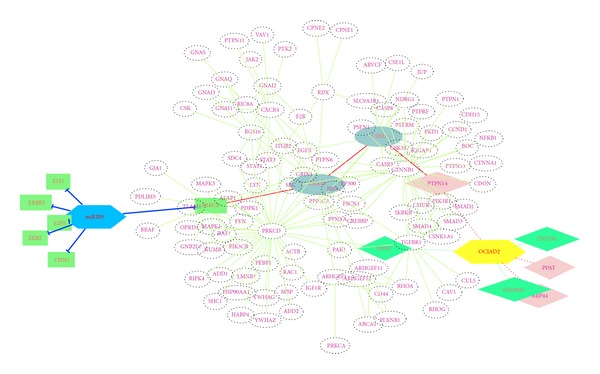


Based on experimental data GDS3634, miR-205 expression effect on prostate cancer cell line, from NCBI public database browser,* P *value of 8 samples of Student's *t*-test, microarray data was applied as the link cost for predicted paths. Searched on the pathway bridge that has been enriched through prostate cancer pathways, the top 10 out of 88 forecasted paths were picked for further biological meaning verification (Supplementary Table 1.2). One reasonable forecast shown in Figure 7 was miR205- PRKCE- CNA13- CDH1- PTPN14-OCIAD2. High correlated genes with OCIAD2 were distinguished as dashed lines (red for positive correlation and green for negative correlation). PRKCE, as one of the six target genes of miR205, was marked as the green box. Among the pathway bridge that consisted of protein nodes (dashed circles) and proteins' interactions (green lines), a shortest protein path with least DES cost was emphasized by red lines.

Suggested by the significant association between TGF*β*1 and CDH1, pbMOO approach was employed again aiming at finding out how TGF*β* affects OCIAD2 across CDH1 in prostate cancer. Interestingly, “TGF*β*1 influenced CDH1 across SMADs” was observed after filtering the predicted protein paths and verified by [17].

#### 3.2.2. Pathways in Liver Cancer

By the analysis of GSE42357 gene expression data, genes like C5, AG7, SDC2, and FHL2 have been suggested to be the candidates of OCIAD2 by their tight correlations with it. Significantly, those candidate genes all play important roles in cancer related processes, for instant, C5 takes the responsibility in inflammatory and cell killing processes [18] and FHL2 acts as both tumor-promoter or tumor-suppressor depending on different types of cancer [19]. The calculated results of the approach were paths with credibility cost, that is, TGF*β*1- TGF*β*R1- CLU- C7- C5-OCIAD2 (cost 0.233181). This pathway was fully explained by the fact that CLU is a modulator of TGF*β*1 signaling pathway by regulating Smad2/3 proteins [20] and the well-known protein interactions CLU-C7 and C7-C5.

### 3.3. Modular Mechanism Exploration

If a signaling transmission process, from extracellular through cytoplasm to nucleus, results in upregulation or downregulation of genes in the cell, then transcription factor (TF) usually plays the downstream role in this signaling flow. Since OCIAD2 was differentially expressed in prostate cancer cell line, liver cancer was associated with mesenchymal stem cells, and especially in TGF*β* treated Panc-1 pancreatic adenocarcinoma cell line, and the question how is OCIAD2 activated by TGF*β* signaling was solved by studying the probable transcription factor of OCIAD2, which also acting as the downstream of TGF*β* signal.

Among all the 30981 genes from Transcriptional Regulatory Element Database [9], 177 transcription factors of homo sapiens were picked out as background human transcription factors library.. The algorithm to find the possible transcription factors of OCIAD2 in TGF*β* treated signaling was divided into three main steps: first, pbMOO approach was employed to calculate the costs of all the shortest paths between TGF*β* and human transcription factors; then the ones with the least costs and high correlations with OCIAD2 in gene expression data were filtered out and selected as candidate transcription factors for OCIAD2; finally, biological TGF*β* induced OCIAD2's differential expression mechanism which was concluded with literature verification.

#### 3.3.1. Speculation of Human TF Enrolled in TGF*β* Signal

As a fresh gene with rare reported property, the discovery of transcription factor in TGF*β*1 signal is the main issue in OCIAD2 study. Among those 177 human transcription factors, the ones with the least pathway cost, which was defined by the sum of gene expression experimental* P *value of proteins on the pathway, are the most credible TFs for OCIAD2. The start point of the pathway was chosen as TGF*β*1, and the end point was OCIAD2. All the pathway costs for those passing through TFs were calculated by applying pbMOO approach and the top of them were listed in the Supplementary Table 1.3.

#### 3.3.2. Feasible TF of OCIAD2 in Cancer Cell Line

In this part, verification of the observation that AR might be the transcription factor of OCIAD2 in TGF*β*1 signal, and SMAD group might enroll this process, was made in the light of gene expression data from both HCC and prostate cancer cell lines.

**Figure 8:**
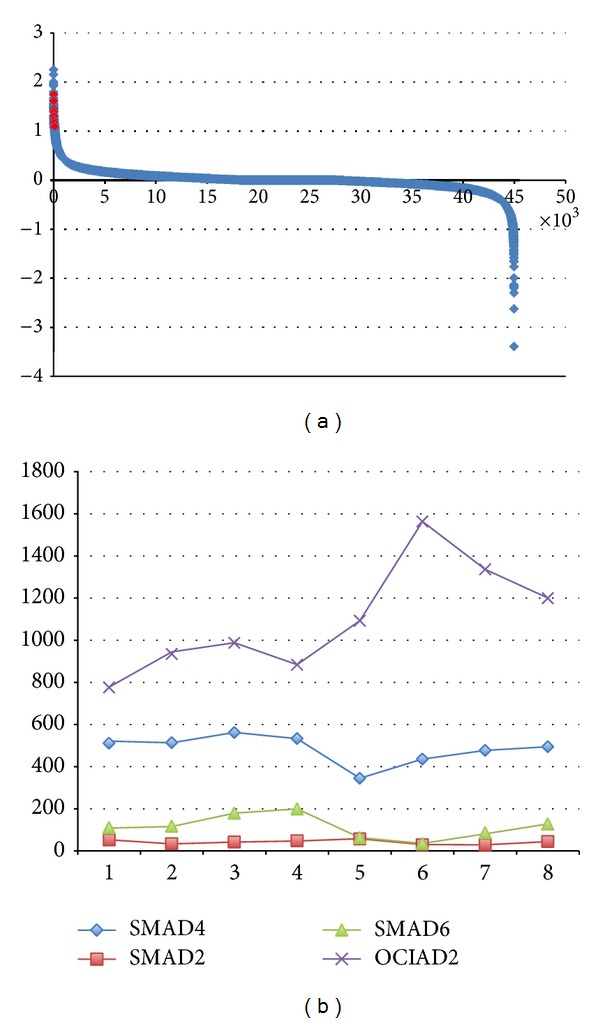


Due to the fact that GSE42357 gene expression data was the comparison between liver cancer associated mesenchymal stem cells (LC8-MSC) and normal ones (LN8-MSC) from the same patient, genes like OCIAD2 had only two experimental values—one for condition LC8-MSC and one for control LN8-MSC. The sample space was too tiny for Pearson Correlation calculation. For better results, the distribution of the fold change of each gene was plotted as the following Figure 8, and evidently, AR, which had ten pairs of experiment data in the range [1.09315, 1.74845], highly differentially expressed in HCC microenvironment. The fold change value of OCIAD2 in the same array data is −0.377255, which implied that AR must have negative effect on OCIAD2, in the other words, it should be the inhibitor of OCIAD2. Not like the top obviously expressed genes with fold changes close to 3.0, the SMAD group showed the relatively lower differential expression—most of them had slight positive changes less than 0.1. However, SMAD4 with fold change 0.06034, SMAD2 0.4908, and SMAD3 0.02102 still survived as pathway proteins in pbMOO predictions, which were ignored by other pathway analysis methods.

Analyzing the results (top 30 were detailed in Supplementary Table 1.3), two interesting facts were observed: AR was shown with the highest frequency as the transcription factor of OCIAD2 in 17 pathways out of the top 50, while STAT5A was the second recurrent one that was the transcription factor of 10 pathways; AR appeared 34 times, and SMAD group proteins appeared 16 times in the top 50 pathways with lowest cost, in which other transcription factors had less occurrences. The observation insinuated that AR was the most reliable transcription factor of TGF*β*1 signal induced OCIAD2, and SMAD group proteins had the closest relationships with this signaling process.

In DU145 prostate cancer cell line with restored miR-205 expression, unfortunately no data mapping with AR was found. However, these 8 samples of experimental data were still powerful to analysis how SMADs enrolled in OCIAD2 expression. As the figure showed, SMAD4 had the largest expression value as well as the highest negative correlation −0.696476 with differential expressed OCIAD2 among SMADs, followed by SMAD2 with correlation value −0.595238 and SMAD6 −0.571429.

#### 3.3.3. Mechanism of TGF*β* Induced OCIAD2's Expression

**Figure 9:**
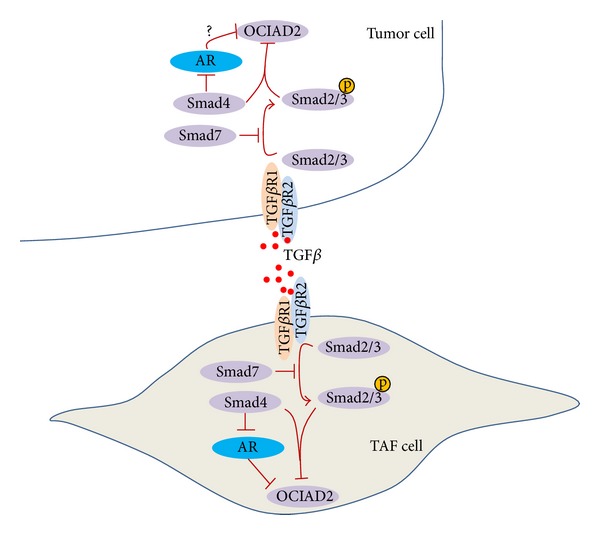


Analyzing the observations comprehensively on modular study and referring to related literature, the signaling pathway from TGF*β*1 targeting OCIAD2 was concluded as shown in Figure 9: the signaling transmits from TGF*β*1- TGF*β*R1- AR-OCIAD2 in liver cancer mesenchymal stem cell, then differentiates into Tumor-Associated-Fibroblasts (TAFs) in tumor stroma. As the only known mammalian coSMAD, SMAD4 transferred signaling from cytoplasm to TGFB signal. AR, the symbol of androgen receptor, mainly functioned as a DNA-binding transcription factor that regulates target gene expression from cytoplasm into nucleus.

Loss of cell adhesions or polarity is widely associated with CDH1 (E-cadherin). This process, referred to as EMT, enhances motility and invasiveness of many cell types and is often considered as a prerequisite for tumor infiltration and migration. TGF*β* mediated induction of EMT processes is associated with specific stages of morphogenesis and during tumorigenesis by activating downstream signaling pathways in both Smad-dependent and Smad-independent mechanisms. The upregulation of OCIAD2 expression by TGF*β* stimulation, and downregulated OCIAD2 expression in metastatic prostate tissues, revealed that OCIAD2 played roles in TGF*β* promoted tumor cell migration, invasion, and mobility. The discovery that OCIAD2 has been enrolled in TGF*β* signal across CDH1 powerfully testified our postulation—OCIAD2 could act as a downstream effector of TGF*β* signals. In our predicted path, SMAD4 had the largest expression value as well as the highest negative correlation −0.696476 with differential expressed OCIAD2 among SMADs families. Previous study reported that Smad3/4 cooperated with Snail1 which acted as corepressors of CDH1 in the EMT process [21]. Due to the lack of information on protein interaction with OCIAD2, future biological assay needs to investigate the potential relationships between CDH1 and OCIAD2 in tumor EMT. In addition, smad signaling is required to maintain epigenetic silencing of some key EMT related proteins in breast cancer progression [22]. Because OCIAD2 frequently methylated in some kinds of cancers [5, 6, 23], we speculate that activated TGF*β*-Smad signaling provides an epigenetic memory to maintain silencing of OCIAD2 in EMT as well. Thus, disruption of TGF*β*-Smad4-OCIAD2 signaling may be a useful therapeutic strategy to target tumor progression.

Specifically, the predicted path TGFB1- TGFBR1- SMAD2/3-SMAD4 was verified by [24, 25], and TGFB1's influence on AR with SMAD3 was also proved in [26].

## 4. Conclusions

In this study, a bioinformatics approach was developed, and it demonstrated that the function-unknown protein ovarian carcinoma immunoreactive antigen-like protein 2 (OCIAD2) is probably regulated by TGF*β* and AR signals in the tumor EMT process. OCIAD2 is an immunoreactive antigen, which functions, involved pathways, and molecular mechanisms have never been reported. Current popular signaling analysis tools like IPA [12] are focusing on the highest differentially expressed genes with sufficient literature supported, ignoring signatures such as OCIAD2. Moreover, as the comprehensive analysis of wide-field database, the output pathways of those tools can hardly be specific for an interesting stimulation or disease. Overcoming the insufficiency of the knowledge on the new gene OCIAD2 and studying the modular signaling mechanism from the given ligand to the pointed signature, the pbMOO approach successfully answered the question “how did the observed signature gene OCIAD2 get involved in ligand TGF*β* stimulation signal,” and detailed the predicted pathways into the tumor microenvironment.

With pbMOO approach, a new pathway “TGF*β*1- TGF*β*R1- AR-OCIAD2” in liver cancer mesenchymal stem cell was predicted, which will differentiate into Tumor-Associated-Fibroblasts (TAFs), one of the major components of tumor stroma. Stromal-epithelial crosstalk regulates all phases of cancer metastasis. In prostate cancer, androgen signaling is central to stromal-epithelial cross-talk in tumor progression. Tissue-based studies of human prostate cancer have shown that stromal AR expression and transcriptional activity downstream of the AR are lower in stromal cells which are derived from carcinomas. Androgen Receptor (AR) may promote hepatocarcinogenesis or suppress HCC metastasis. These opposite roles of AR also occur in prostate cancer [27]. The potential mechanisms for the AR dual roles are possibly caused by the differential AR signals in different cellular types having an oncogenic role in stroma and epithelial cells, but a suppressive role in basal intermediate epithelial cells. As DU-145 is an AR-independent cell lacking AR protein expression, the predicted path from AR to OCIAD2 in prostate cancer needs more support of more biological experiments. Further biological experiments are still needed to explore the existence of pathway TGF*β*-Smad4-OCIAD2 signaling in AR-dependent cell models as well.

The signal from TGF*β*, via the AR, played a critical role in the deregulation of TGF*β* signaling in prostate and/or liver tumorigenesis, and those TGF*β* effectors (Smads 3 and 4) serving as negative regulators of AR-mediated transcription in cancer cells have been established by several investigations [28]. With pbMOO approach, the functional unknown protein OCIAD2 was also enrolled into a signal pathway “TGF*β*1- TGF*β*R1- SMAD2/3- SMAD4- AR-OCIAD2” in tumor and adjacent microenvironment. Currently, clinical studies using antiandrogens had disappointing results, few beneficial effects on patients, or even less survivals. Understanding the molecular mechanisms of AR in tumor microenvironment will undoubtedly further improve the results obtained with antitumor therapeutic strategies.

## Supplementary Material

Supplementary 1.1: The figure showed the whole FANMOD analysis of the occurring frequency of all four-vetex motifs in the protein-to-protein network. Motifs ID 31710, 13278, 4958 and 27030 all had tiny frequency less than 5%, positive Z-score and zero p-value, which means they happened in smaller probabilities than the other structures in this network. Other than umbrella-looked ID 4382 and chain-looked ID 8598, the previous four motifs shared a common character—looping structure. Hence, the whole protein-to-protein network can be refined by triangle and rectangle structures, which are the only two possible looping elements of four-vetex motifs.
Supplementary 1.2: The table listed the top 30 predicted protein paths connecting miR205 and OCIAD2 ranked by the costs. The prediction cost was defined as the p-values summation of the proteins on the path. As p-value represents the probability that a random path has more occurring than the predicted one, the prediction with less cost was more reliable than others. However, due to the fact that the protein-to-protein interactions are undirected, calculation-based predictions might have paths with wrong directions that will never happen in the real biomedical world. Filtering followed this calculated result by deleting those bio-meaningless pathways.
Supplementary 1.3: In order to find the transcription factor of OCIAD2, the 177 human transcription factors were tried as the direct upstream of OCIAD2 one by one, and all the possible pathways were calculated and ranked by the prediction cost. The table showed the most possible 30 pathways. AR appeared in 19 out of the top 30 predictions; among them, AR worked as transcription factor of OCIAD2 for 9 times, followed by STAT5A with 3 times. Hence, among all the 177 human transcription factors, AR might be the most possible one for OCIAD2 in TGF*β* signal.

## References

[1] Witz IP, Levy-Nissenbaum O (2006). The tumor microenvironment in the post-PAGET era. *Cancer Letters*.

[2] Zeisberg M, Hanai JI, Sugimoto H (2003). BMP-7 counteracts TGF-*β*1-induced epithelial-to-mesenchymal transition and reverses chronic renal injury. *Nature Medicine*.

[3] Kojima Y, Acar A, Eaton EN (2010). Autocrine TGF-*β* and stromal cell-derived factor-1 (SDF-1) signaling drives the evolution of tumor-promoting mammary stromal myofibroblasts. *Proceedings of the National Academy of Sciences of the United States of America*.

[4] Luo LY, Soosaipillai A, Diamandis EP (2001). Molecular cloning of a novel human gene on chromosome 4p11 by immunoscreening of an ovarian carcinoma cDNA library. *Biochemical and Biophysical Research Communications*.

[5] Gueugnon F, Leclercq S, Blanquart C (2011). Identification of novel markers for the diagnosis of malignant pleural mesothelioma. *The American Journal of Pathology*.

[6] Kulis M, Heath S, Bibikova M (2012). Epigenomic analysis detects widespread gene-body dna hypomethylation in chronic lymphocytic leukemia. *Nature Genetics*.

[7] Noushmehr H, Weisenberger DJ, Diefes K (2010). Identification of a cpg island methylator phenotype that defines a distinct subgroup of glioma. *Cancer Cell*.

[8] Kim H, Watkinson J, Varadan V, Anastassiou D (2010). Multi-cancer computational analysis reveals invasion-associated variant of desmoplastic reaction involving INHBA, THBS2 and COL11A1. *BMC Medical Genomics*.

[9] Jiang C, Xuan Z, Zhao F, Zhang MQ (2007). TRED: a transcriptional regulatory element database, new entries and other development. *Nucleic Acids Research*.

[10] Peri S, Navarro JD, Kristiansen TZ (2004). Human protein reference database as a discovery resource for proteomics. *Nucleic Acids Research*.

[11] Kanehisa M, Goto S (2000). Kegg: kyoto encyclopedia of genes and genomes. *Nucleic Acids Research*.

[12] http://www.ingenuity.com/.

[13] Wernicke S, Rasche F (2006). FANMOD: A tool for fast network motif detection. *Bioinformatics*.

[14] Jin G, Cui K, Zhou X, Wong STC (2009). Unraveling the signal-transduction networks in cancer metastasis. *IEEE Signal Processing Magazine*.

[15] Schreiber F, Schwöbbermeyer H Motifs in biological networks.

[16] Ogawa M, LaRue AC, Drake CJ (2006). Hematopoietic origin of fibroblasts/myofibroblasts: its pathophysiologic implications. *Blood*.

[17] Stegmüller J, Huynh MA, Yuan Z, Konishi Y, Bonni A (2008). TGF*β*-Smad2 signaling regulates the Cdh1-APC/SnoN pathway of axonal morphogenesis. *Journal of Neuroscience*.

[18] Varani J, Bendelow MJ, Sealey DE (1988). Tumor necrosis factor enhances susceptibility of vascular endothelial cells to neutrophil-mediated killing. *Laboratory Investigation*.

[19] Amann T, Egle Y, Bosserhoff A-K, Hellerbrand C (2010). FHL2 suppresses growth and differentiation of the colon cancer cell line HT-29. *Oncology Reports*.

[20] Lee KB, Jeon JH, Choi I, Kwon OY, Yu K, You KH (2008). Clusterin, a novel modulator of TGF-*β* signaling, is involved in Smad2/3 stability. *Biochemical and Biophysical Research Communications*.

[21] Vincent T, Neve EPA, Johnson JR (2009). A Snail1-Smad3/4 transcriptional repressor complex promotes TGF-*β* mediated epithelial-mesenchymal transition. *Nature Cell Biology*.

[22] Papageorgis P, Lambert AW, Ozturk S (2010). Smad signaling is required to maintain epigenetic silencing during breast cancer progression. *Cancer Research*.

[23] Matsumura S, Imoto I, Kozaki K (2012). Integrative array-based approach identifies mzb1 as a frequently methylated putative tumor suppressor in hepatocellular carcinoma. *Clinical Cancer Research*.

[24] Berndt SI, Huang WY, Chatterjee N (2007). Transforming growth factor beta 1 (TGFB1) gene polymorphisms and risk of advanced colorectal adenoma. *Carcinogenesis*.

[25] Lan HY (2011). Diverse roles of TGF-*β*/smads in renal fibrosis and inflammation. *International Journal of Biological Sciences*.

[26] Kang HY, Lin HK, Hu YC, Yeh S, Huang KE, Chang C (2001). From transforming growth factor-*β* signaling to androgen action: Identification of Smad3 as an androgen receptor coregulator in prostate cancer cells. *Proceedings of the National Academy of Sciences of the United States of America*.

[27] Niu Y, Altuwaijri S, Lai KP (2008). Androgen receptor is a tumor suppressor and proliferator in prostate cancer. *Proceedings of the National Academy of Sciences of the United States of America*.

[28] Kang HY, Huang KE, Chang SY, Ma WL, Lin WJ, Chang C (2002). Differential modulation of androgen receptor-mediated transactivation by smad3 and tumor suppressor smad4. *Journal of Biological Chemistry*.

