# Supplementary material for: Pathway Bridge Based Multiobjective Optimization Approach for Lurking Pathway Prediction
Source: Biomed Res Int. 2014 Apr 16;2014:351095. doi: 10.1155/2014/351095 (PMC4052696; doi:10.1155/2014/351095)
Supplement: Supplementary file 1 — Supplementary 1.1: The figure showed the whole FANMOD analysis of the occurring frequency of all four-vetex motifs in the protein-to-protein network. Motifs ID 31710, 13278, 4958 and 27030 all had tiny frequency less than 5%, positive Z-score and zero p-value, which means they happened in smaller probabilities than the other structures in this network. Other than umbrella-looked ID 4382 and chain-looked ID 8598, the previous four motifs shared a common character—looping structure. Hence, the whole protein-to-protein network can be refined by triangle and rectangle structures, which are the only two possible looping elements of four-vetex motifs. Supplementary 1.2: The table listed the top 30 predicted protein paths connecting miR205 and OCIAD2 ranked by the costs. The prediction cost was defined as the p-values summation of the proteins on the path. As p-value represents the probability that a random path has more occurring than the predicted one, the prediction with less cost was more reliable than others. However, due to the fact that the protein-to-protein interactions are undirected, calculation-based predictions might have paths with wrong directions that will never happen in the real biomedical world. Filtering followed this calculated result by deleting those bio-meaningless pathways. Supplementary 1.3: In order to find the transcription factor of OCIAD2, the 177 human transcription factors were tried as the direct upstream of OCIAD2 one by one, and all the possible pathways were calculated and ranked by the prediction cost. The table showed the most possible 30 pathways. AR appeared in 19 out of the top 30 predictions; among them, AR worked as transcription factor of OCIAD2 for 9 times, followed by STAT5A with 3 times. Hence, among all the 177 human transcription factors, AR might be the most possible one for OCIAD2 in TGFβ signal. [file 351095.f1.pdf]

Supplementary of Pathway Bridge Based Multi-objective Optimization Approach for Lurking Pathway Prediction

Rengjing Zhang, Chen Zhao, Zixiang Xiong, Xiaobo Zhou

October 14, 2013

1 Supplementary

1.1 Motif Detection of PPI Network by FANMOD Tool

ID	Adj	Frequency [Original]	Mean-Freq [Random]	Standard-Dev [Random]	Z-Score	p-Value
31710	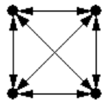	0.017858%	1.1634e-007%	1.6208e-008	11018	0
13278	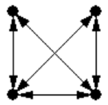	0.27748%	6.9157e-005%	4.1539e-006	667.84	0
4958	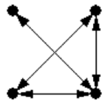	4.7955%	0.0085102%	0.00042599	112.37	0
4382	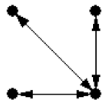	50.482%	54.627%	0.0017771	-23.325	1
27030	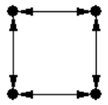	0.31215%	0.24795%	7.7688e-005	8.264	0
8598	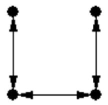	44.115%	45.117%	0.0016806	-5.9588	1

Figure 1: Four-vetex Motifs of PPI network by FANMOD Outputs

1.2 Raw Pathway Prediction Table for Prostate Cancer

Possible Protein Paths from miR205 to OCIAD2 in Prostate Cancer

No.								Cost[1]
1	PTPN14	CDH1	G-113	PRKCE				0.152141
2	PTPN14	TJP1	GRIN1	PRKCE				0.165309
3	PTPN14	YWHAG	TIAM1	PRKCE				0.182662
4	PTPN14	YWHAG	SRC	PRKCE				0.403788
5	PTPN14	JUP	SRC	PRKCE				0.505998
6	TAL2	MAPK3	PRKCE					0.513332
7	CAPN2	NFKBIA	CUL1	E2F1				0.513761
8	PKP3	DSC2	JUP	SRC	PRKCE			0.571772
9	CAPN2	NMT1	LYN	PLCG1	GRIN1	PRKCE		0.581042
10	CAPN2	NFKBIA	SRC	PRKCE				0.671004
11	PPP2R1B	RELA	COMMD1	CUL1	E2F1			0.680008
12	MYD88	IRAK2	HRAS	GRIN1	PRKCE			0.755849
13	PPP2R1B	RELA	HDAC3	CCND1	NPDC1	E2F1		0.761167
14	CAPN2	CDK5R1	CHN1					0.775821
15	PPP2R1B	SET	KLF5	PRKCD	YWHAG	MDM4	E2F1	0.786134
16	PPP2R1B	PPP2CA	CCNG1	CDK5	CABLES1	CC-11	E2F1	0.80783
17	PPP2R1B	RELA	PARP1	E2F1				0.817619
18	MYD88	IL1RAP	RAC1	CHN1				0.833815
19	MYD88	IL1RAP	RAC1	TIAM1	PRKCE			0.871396
20	PPP2R1B	SET	SGOL1	XRCC6	CC-11	E2F1		0.903496
21	PPP2R1B	RELA	HDAC3	NRIP1	VDR	CDK7	E2F1	0.92203
22	PTPN14	CDH1	IQGAP1	MAPK1	PRKCE			0.937152
23	MYD88	IRAK1	PRKCI	SRC	PRKCE			0.949189
24	PTPN14	CDH1	IRS1	MAPK1	PRKCE			0.980665
25	PPP2R1B	RELA	MYC	GSK3B	E2F1			0.997489
26	TAL2	MAPK3	MYC	RBL1	E2F5			1.011626
27	CAPN2	NMT1	LYN	MAPK3	PRKCE			1.026447
28	PPP2R1B	HDAC1	PHB	E2F1				1.030947
29	TAL2	TCF3	CREBBP	E2F1				1.047172
30	PPP2R1B	RELA	HDAC3	PPARG	NCOA6	E2F1		1.048389
⋮	⋮	⋮	⋮	⋮	⋮	⋮	⋮	⋮

1.3 Prediction Table of Potential Transcription Factor

OCIAD2's Transcription Factors Detection in TGFβ Signal

No.									Cost
1	TGFB1	ITGAV	ITGB1	ITGA3	FHL2	AR	OCIAD2		0.022027
2	TGFB1	ITGAV	ITGB1	FHL2	AR	OCIAD2			0.024967
3	TGFB1	THBS1	PDGFB	PDGFRB	STAT4A	OCIAD2			0.029893
4	TGFB1	THBS1	SPARC	PLAT	MAPK3	CAV1	AR	OCIAD2	0.033335
5	TGFB1	VTN	FGF2	CASP1	AR	OCIAD2			0.035584
6	TGFB1	VTN	SERPINE1	KRT18	RAF1	AR	OCIAD2		0.036216
7	TGFB1	ACVRL1	XIAP	TRAF6	CAV1	AR	OCIAD2		0.047305
8	TGFB1	THBS1	LRP5	SMAD9	OCIAD2				0.062151
9	TGFB1	DCN	EGFR	AR	OCIAD2				0.063297
10	TGFB1	TGFBR1	CAV1	AR	TRIM24	RXRA	PPARG	OCIAD2	0.078664
11	TGFB1	TGFBR1	CAV1	AR	PNRC1	RARG	OCIAD2		0.081610
12	TGFB1	TGFBR1	CAV1	AR	TRIM24	RXRA	PPARG	OCIAD2	0.081804
13	TGFB1	TGFBR1	CAV1	AR	FHL2	WT1	OCIAD2		0.082158
14	TGFB1	TGFBR1	SMAD7	PIAS1	AR	OCIAD2			0.083669
15	TGFB1	TGFBR1	SMAD7	PIAS1	MSX1	OCIAD2			0.08367
16	TGFB1	TGFBR1	SMAD7	HEYL	SMAD9	OCIAD2			0.083735
17	TGFB1	TGFBR1	PIK3R1	PDGFRB	STAT5A	OCIAD2			0.083851
18	TGFB1	TGFBR1	PIK3R1	IL2RB	STAT5A	OCIAD2			0.083851
19	TGFB1	TGFBR1	CAV1	AR	OCIAD2				0.086596
20	TGFB1	TGFBR1	CAV1	MAPK3	STAT5A	OCIAD2			0.087053
21	TGFB1	TGFBR1	CAV1	AR	HIF1A	OCIAD2			0.08864
22	TGFB1	TGFBR1	SMAD7	OCIAD2					0.089536
23	TGFB1	TGFBR1	SMAD6	OCIAD2					0.089536
24	TGFB1	TGFBR1	CTNNB1	AR	OCIAD2				0.09008
25	TGFB1	TGFBR1	CAV1	AR	NCOA1	FOS	USF2	OCIAD2	0.090869
26	TGFB1	TGFBR1	CAV1	AR	NCOA1	FOS	OCIAD2		0.093771
27	TGFB1	TGFBR1	CTNNB1	HIF1A	OCIAD2				0.095064
28	TGFB1	TGFBR1	CTNNB1	MITF	FOS	OCIAD2			0.099917
29	TGFB1	TGFBR1	PAK1	CASP1	AR	OCIAD2			0.10004
30	TGFB1	TGFBR1	CAV1	AR	FOXO1	PPARG	OCIAD2		0.106862
⋮	⋮	⋮	⋮	⋮	⋮	⋮	⋮	⋮	⋮

[1]The less Cost, the more credible the pathway is.
